# Drought Tolerant Index and Heterosis Level of Soybean {*Glycine max* (L.) Merrill} Genotypes

**DOI:** 10.1155/sci5/1213004

**Published:** 2025-03-13

**Authors:** Made J. Mejaya, Suhartina Suhartina, Purwantoro Purwantoro, Rudy Soehendi, Trustinah Trustinah, Febria C. Indriani, Gatut W. A. Susanto, Yuliantoro Baliadi, Apri Sulistyo, Sholihin Sholihin, I. GKD. Arsana, Ratna W. Arief, Robet Asnawi, Arief Harsono

**Affiliations:** ^1^National Research and Innovation Agency, Jl. MH Thamrin No. 8, Kota Jakarta Pusat 10340, Jakarta, Indonesia; ^2^Agricultural Instruments Standardization Agency, Jl. Raya Ragunan No. 29 Jati Padang, Pasar Minggu, Jakarta Selatan 12540, Jakarta, Indonesia

**Keywords:** drought tolerant, heterosis, reproductive stage, seed yield, soybean

## Abstract

Drought stress on soybean crops in some areas becomes more severe due to climate change. The objective of this study was to estimate the drought stress tolerant index (STI) and heterosis level of soybean genotypes derived from crosses of Grobogan (G) and Dering 1 (D) varieties. Field research was conducted in Probolinggo, East Java, Indonesia, during late dry season of 2016. A total of 17 soybean genotypes consisted of 15 lines derived from crosses of G × D varieties, and two check varieties (Grobogan and Dering 1) were tested using a randomized block design and repeated three times. Each genotype was grown in two environments, namely, optimal irrigation condition (irrigation applied in planting until ripening pods) and drought condition in the reproductive phase (irrigation applied in planting until flowering). Results of the study showed that in drought condition, line (G/D) -99-32-14 had the highest seed weight/plant, highest STI, and highest both heterosis and heterobeltiosis value base on seed weight/plant. In drought condition, most of the soybean lines showed positive heterosis values on 100 seed weight. The cross of Dering 1 and Grobogan varieties produced one soybean line (G/D) -99-32-14 which was more tolerant to drought and had seed size (100 seed weight) higher than Dering 1 (the current drought tolerance variety). It is concluded that it is possible to obtain soybean genotype tolerant to drought having higher seed yield and medium seed size than the better parent.

## 1. Introduction

Most of the soybean {*Glycine max* (L.) Merrill} in Indonesia are planted during dry season in rainfed land with limited water irrigation. This condition causes the crop failure to grow due to drought. The effect of drought in some areas becomes more severe due to climate change, including soybean plants. The development of drought tolerant and stable genotypes across the environments is necessary since there is wide range of variation in the varietal performance in diverse environments. Drought stress in 50% of available water could reduce soybean seed yield more than 50%. Due to water shortage of 70% field capacity during generative phase, 15 soybean genotypes showed grain yield of 8.40 g/plant compared to 14.31 g/plant in optimal water condition of 100% field capacity. Due to water shortage of 20%–30% field capacity during generative phase, DV/2984-330 soybean line showed grain yield of 1.95 t/ha out of its yield potential of 2.83 t/ha [1, 2].

Drought and heat affect the seed yields by reducing yield components such as seed size and seed number as well as seed quality [3]. Availability of varieties that are more tolerant to drought and large seed size than the existing varieties is necessary. Drought-tolerant soybean varieties need to be done sustainably to increase national soybean productivity and production.

In soybean, plant breeding is the process of selecting the parental combinations to produce progeny superior to either parent. Drought response is defined as the relative yield of a certain genotype compared to other genotypes which are subjected to the same drought stress. The stress tolerance index, abbreviated as STI, could be used as a tool for screening drought tolerant genotypes [4]. Soybean tolerance mechanism to drought has an important role, because it relates to characters that support the tolerance. In soybean breeding, the combination of physiological, morphological, and agronomical characters is necessary, because these three characters usually are not genetically linkage. By combining these three characters, decreasing yield due to drought stress can be repressed.

To measure the improvement derived from soybean parent crosses, a heterosis STI value is used in field test. The existence of heterosis in some soybean crosses could be a useful criterion for selection among biparental crosses [5, 6]. Expected value of heterobeltiosis and heterosis is needed in order to assess the economic feasibility [7].

Soybean breeder at the Indonesian Legumes and Tuber Crops Research Institute (ILETRI) has developed 15 promising lines that need to be tested for their tolerant to drought performance and seed size compared to existing varieties. The crosses of Grobogan variety (large seed size) and Dering 1 variety (drought tolerant) as parents were conducted to obtain variety having large seed size and drought tolerant. Dering 1 variety is tolerant to water shortage up to 30% of field capacity with high yield potential [8]. Grobogan is not drought resistance variety especially during the reproductive phase; however, this variety has high seed yield potential (3.40 t/ha) and large seed size (18 g/100 seeds) (Table 1) [9].

The selection of drought stress during the reproductive stage or phase of soybean lines had been carried out in generations F3–F5, and then, the results were tested in the preliminary and advance yield tests. The objective of this study was to estimate the drought STI and heterosis level of soybean genotypes derived from crosses of Grobogan and Dering 1 varieties.

## 2. Material and Methods

### 2.1. Study Sites

The field research was conducted during late dry season of 2016 at Muneng Experimental Station (7° 48′ 7.2″S and 113° 9′ 32.4″E; 10 meters above sea level), Probolinggo, East Java, under the ILETRI, Malang, East Java, Indonesia. There was no rainfall occurred during the field study. Soil water content was measured during the reproductive phase, when plants began flowering to physiological maturity phases at intervals of 7–10 days using gravimetric methods and soil water content at pF 0, 2.0, 3.0, and 4.2 before planting (in Soil Physics Laboratory, Faculty of Agriculture, Brawijaya University, Malang, East Java, Indonesia). The groundwater content measured in the field at pF 0, 2.0, 2.5, 3.0, and 4.2 was 54.0%, 50.0%, 47.0%, 39.0%, and 25.0%, respectively. The groundwater content in the field capacity condition (pF 2.5) at the Muneng location was 47.0%, while at the permanent wilting point (pF 4.2), it was 25.0%, with a percentage of available water at 22.0%. Field condition showed that in Muneng Experimental Station, the selection objective to obtain drought tolerant lines can be achieved.

### 2.2. Plant Materials

A total of 17 soybean genotypes consisted of 15 lines derived from crosses of Grobogan (G) variety × Dering 1 (D) variety and two parents (Grobogan and Dering 1). The Grobogan and Dering 1 were used as parents due to the fact that Grobogan variety has large seed size but susceptible to drought; meanwhile, Dering 1 has drought tolerant response but small seed size. By making the crosses of these two varieties, it is expected to obtain variety having large seed size and drought tolerant. The characteristics of Grobogan and Dering 1 varieties are presented in Table 1.

**Figure 1:**
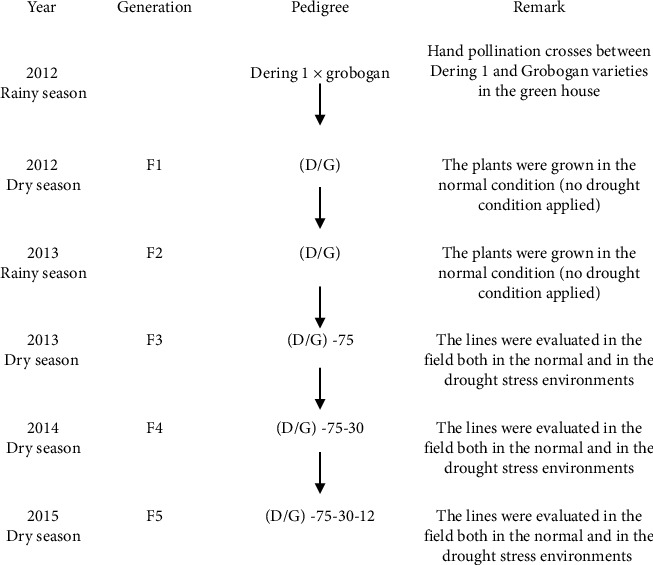
The pedigree of line (D/G) -75-30-12 derived from selection of crosses between Grobogan and Dering 1 of drought tolerance soybean.

The pedigree of line (D/G)-75-30-12 as an example which was derived from the selection of crosses between Grobogan and Dering 1 of soybean drought tolerance is shown in Figure 1. The crosses between Grobogan and Dering 1 were conducted in 2012 in the green house. During F1–F2 generations, the plants were grown in the normal condition (no drought condition applied). During F3–F5 generations, the lines were evaluated both in the normal condition and in a drought stress environments during the reproductive phase. The selection was carried out until the F5 and drought lines were obtained.

The selection method used was a combination of bulk and pedigree methods. Generation F1 and F2 were planted in bulk, namely, in greenhouses (F1) on rainy season 2012 and in the field (F2) on dry season 2012. The selection of pedigrees began in the F3 generation on dry season 2013, F4 on dry season 2014, and F5 on dry season 2015. In 2015, a total of 15 F5 lines having drought tolerance and early maturity were obtained through a pedigree selection. The selection of the F3–F5 lines was carried out based on maturity and good plant performance (lots of pods, deterministic growth type, and shorter maturity than both parents (Dering 1 and Grobogan varieties) were planted as material for selection F4). The selection of the F5 generation was conducted based on the lines having very good plant performance, where in the stress condition by which in the limited water, the plants still grew and produced the seeds. The selection criteria to obtain a drought stress tolerant genotype were conducted using STI [10].

### 2.3. Implementation of the Study

A total of 17 soybean genotypes were tested using a randomized block design with three replications. Each genotype was grown in two environments, namely, optimal irrigation condition (irrigation applied in planting until ripening pods) and drought condition in the reproductive phase (irrigation applied in planting until flowering). Each genotype was planted on a plot of 2.2 m wide and 3.3 m long, with plant spacing of 40 cm × 15 cm, two plants per hole. Petroganik fertilizer of 1 ton/ha was given before planting by spreading it out, whereas Phonska fertilizer of 250 kg/ha and SP36 of 100 kg/ha were given during planting also by spreading it out. Pest and disease control was carried out preventively by using chemical pestisida according to pest conditions in the field with dosage as recommended.

### 2.4. Data Observation

Observations conducted include number of normal or filled pods per plant, weight of 100 seeds, and seed weight per plant were performed on five sample plants. Statstical software used was PB Tools: Software for Plant Breeders.

### 2.5. Data Analysis

The selection criteria for selecting a drought tolerant genotype were based on the STI formula developed by Fernandez [10] and Yahoueian et al. [4]. They defined tolerance STI to identify genotypes that produce high seed weight under both nonstress stress (optimal irrigation) and stress conditions (limited irrigation) environment. The higher the STI value of a genotype, the more tolerant the genotype is to stress.

(1) $$\begin{array}{c} \left(\text{STI}\right)=\frac{Y_{p}\times Y_{s}}{{\left(\overset{¯}{Y_{p}}\right)}^{2}}, \end{array}$$

where *Y*_*s*_ is seed weight of each genotype in stress conditions, *Y*_*p*_ is seed weight of each genotype in nonstress conditions, and $\overset{¯}{Yp}$ is mean seed weight in nonstress condition for all genotypes.

At normal irrigation and drought stress conditions, each line of the Dering-1 (P1) and Grobogan (P2) crosses and percent heterosis (H) and heterobeltiosis (Hb) for each cross (hybrid) are calculated by the following formula described by Syukur et al. [11]:

(2) $$\begin{array}{c} H\left(\%\right)=\left(\frac{\left(F1-\text{MP}\right)}{\text{MP}}\right)x100\%, \end{array}$$

(3) $$\begin{array}{c} \text{Hb}=\left(\frac{\left(F1-\text{HP}\right)}{\text{HP}}\right)x100\%, \end{array}$$

where *F*1 = observations of F1 from the total *F*1 plants; MP = average observed value of both parents (*P*1 and *P*2); HP = better parental observation value (*P*1 or *P*2).

## 3. Results and Discussion

### 3.1. Drought Effect on the Seed Weight/Plant

The seed weight/plant of 17 genotypes in normal irrigation ranged from 9.60 g to 14.25 g with average of 11.34 g, while in drought condition, the seed weight/plant ranged from 4.78 g to 9.48 g with average of 6.99 g. There was a 38% reduction on seed weight/plant due to drought stress (Table 2). This finding which is lower than the result of 51%–63% was reported by Suhartina [1] and Suhartina and Kuswantoro [2]. The drought STI of seed weight/plant ranged from 0.34 (Grobogan) to 0.86 ((G/D) -99-32-14) with the mean of 0.62. There were nine lines derived from crosses of Grobogan × Dering had STI values above the check drought tolerance variety Dering 1 (0.60), and all 15 lines had STI values above the check drought susceptible variety Grobogan (0.34) (Table 2).

The heterosis level of seed weight/plant in normal irrigation ranged from 9% to 62% with average of 33.0%, while in drought condition, the heterosis ranged from −30%–39% with average of 33.0%. The heterosis level of STI base on seed weight/plant in normal irrigation ranged from −21% −0.05%–80% with the average of 32.8%. The heterobeltiosis level of seed weight/plant in normal irrigation ranged from 9% to 54% with average of 26.3%, while in drought condition, the heterobeltiosis ranged from −43% to 13% with average of −16.5% (Table 2).

The heterobeltiosis level of STI base on seed weight/plant in normal irrigation ranged from −36% to 44% with the average of 6.2%. In normal irrigation, genotipe number 1 which is line (G/D) -75-30-12 had the highest seed weight/plant (14.25 g), highest heterosis, and highest heterobeltiosis level base on seed weight/plant. In drought condition, genotipe number 6 or (line (G/D) -99-32-14) had the highest seed weight/plant (9.48 g), highest STI (0.86), highest heterosis (39%), and highest heterobeltiosis (13%) level base on seed weight/plant (Table 2). Positive heterosis values of seed yield/plant also reported by Nassar [12] that seven and four crosses exhibited highly significant favorable positive to mid and better parent (heterobeltiosis) value, respectively, by which the cross (L86-K-73 XH155) exhibited the best heterosis for midparents and better parent of 98.79% and 42.52%, respectively. Similar result was reported by Pereira, Vello, and Rocha [13] that the heterosis of three crosses on soybean grain yield was extremely high (98%). Higher-parent heterosis (Heterobeltiosis) in soybean single crosses for grain yield ranged from minus 41.11%–11.19% [14].

### 3.2. Drought Effect on the 100 Seed Weight

Drought effect on the 100 seed weight is shown in Table 3. Drought condition affected the 100 seed weight as shown by average of 15.71 and 12.73 pods/plant in normal irrigation and drought condition, respectively, with STI value of 0.82. The 100 seed weight of 17 genotypes in normal irrigation ranged from 11.77 g (Dering 1) to 21.93 g (Grobogan), while in drought condition, the 100 seed weight ranged from 8.52 (Dering 1) to 14.53 g ((D/G) -245-47-28). The STI values ranged from 0.41 (Dering 1) to 1.28 (Grobogan), with the mean of 0.82.

The heterosis level of 100 seed weight in normal irrigation ranged from −16% to 3% with average of −8.0%, while in drought condition, the heterosis ranged from −2–27% with average of 12.5%. The heterosis level of STI base on 100 seed weight in normal irrigation ranged from −23% to 12% with the average of −3.2%. The heterobeltiosis level of seed weight/plant in normal irrigation ranged from −36% to −21% with average of −29.1%, while in drought condition, the heterobeltiosis ranged from −22% to 1% with average of −10.4%. The heterobeltiosis level of STI base on 100 seed weight ranged from −50% to 26% with the average of −36.5%. The negative values of heterobeltiosis suggested that there was no soybean lines derived from crosses of Grobogan × Dering had 100 seed weight higher than o the check drought susceptible variety Grobogan (Table 3).

In drought condition, most of the soybean lines showed positive heterosis values, suggested that these lines had 100 seed weight higher than the midparent (Dering 1 and Grobogan). However, all of soybean lines showed negative heterobeltiosis values, suggested that all lines had 100 seed weight lower than the better parent (Grobogan) or in other words, and there was no line had 100 seed weight higher than the better parent (Grobogan). As for 100 seed weight, significant positive heterotic effects were also detected for seven and three crosses relative to midparents and better parent, respectively [12].

### 3.3. Drought Effect on the Pods Number/Plant

Drought effect on the pods number/plant is shown in Table 4. Drought condition did not affect the total pods number/plant as shown by average of 45.5 and 46.7 pods/plant in normal irrigation and drought condition, respectively, with STI value of 1.03. However, drought condition affected the normal pods number/plant and empty pods number/plant. The average of normal pods number/plant was 41.5 and 34.8 pods in normal irrigation and drought condition, respectively, with STI value of 0.84 while the average of abnormal (empty) pods number/plant was 4.0 and 11.9 pods in normal irrigation and drought condition, respectively, with STI value of 2.97. There were four lines derived from crosses of Grobogan × Dering had STI values of normal pods number/plant equal to the check drought tolerance variety Dering 1 (1.21) (Table 4).

In normal irrigation condition, 14 of 15 soybean lines showed positive heterosis values of normal pods number/plant ranged from 1% to 23% except line ((D/G) -75-30-12) with average of 12%. While in drought condition, 8 of 15 soybean lines showed positive heterosis values, suggested that most of these 15 lines had normal pods number/plant higher than the midparent (Dering 1 and Grobogan). Drought is one of the most important factors that limits N fixation, growth, and yield of soybean. Water stress decreased bacteria activity, number of pods per plant, number of seeds per pod, seed weight, and seed yield in drought susceptible genotypes [15] while genotypes identified as relatively drought tolerant lines mostly showed less reduction for number of pods per plant, seed yield, and hundred seed weight under induced drought conditions [16]. The heterotic crop phenotype provides genetic control in designing a high-yielding crop plant integrated with the seed embryo, through plant morphogenesis, homeostatic photosynthesis, and the photobiological sensory regulation of the plant related to stress conditions in the field [17].

Positive heterosis values of normal pods number/plant are also reported by Nassar [12]. That number of pods/plant of all crosses showed a highly significant positive heterosis percentage relative to midparent and better parent. The heterotic effects of hybrids are affected by the diversity of geographical of parents origin. [18]. Expected value of heterobeltiosis, heterosis, and the gene action is needed to know to optimize the development strategy of soybean varieties. The soybean yield is controlled by dominant genes and over-dominant. Combination crosses Willis × MLG 0706 (reciprocal) have a higher potential ratio for die character of the number of pods and seeds yield/plant compared to the value of the other potence ratio. The development of soybeans with the use of hybrid heterosis phenomenon still needs to be assessed in terms of economic feasibility [8].

Friedrichs et al. [19] reported thirty‐seven F_6:7_ inbred lines from each of five biparental populations and obtained the midparent yield heterosis of 15% with significant genetic variation among inbred soybean lines derived from a single cross. The average yield of Cross 1 F1 was 16% greater than that of the highest yielding parent and the average yield of the Cross 2 F1. The magnitude of yield heterosis may be a useful criterion for selection among biparental crosses, and the existence of heterosis was an evidence that superior gene combinations are possible [6]. Heterosis of 21 soybean hybrids was significant positive in 16 hybrids over midparent and in 9 hybrids over better parent. Heterosis mean values were 32% for seed yield/plant, 28% for pods number/plant, 8% for primary branches number/plant, and 2% for 100 seeds weight. Heterosis for seed yield was generally accompanied by heterosis for yield components [20].

Sixteen soybean hybrids were tested, and significant positive heterosis over midparent was recorded in five crosses while positive heterosis over better parent was observed in four crosses for grain yield per plant. It is primarily due to complementary combination of component traits such as plant dry weight, harvest index, number of pods per plant, and number of branches per plant. These heterotic cross combinations could be exploited to get superior segregants of midparents and better parents [21, 22].

The highest heterobeltiosis value was derived by crosses of Willis × Grobogan, by which these crosses have the 58.31% higher seed yield than the best parents [8]. Heterosis in soybean crosses also occurred in total isoflavone (TIF) concentration. Bi et al. [23] reported that positive general combining ability (GCA) effects and heterosis in hybrids which had relative higher TIF concentration level parents showed better performance than those which had lower TIF concentration level parents. Isoflavone content was mainly determined by additive type of gene action that indicated the potential hybrid for isoflavone content improvement.

From the current study, it is obtained that the seed yield mean and its yield components of the crosses were higher than the parent means, indicating that it is possible to obtain progenies or genotypes superior to the parents for grain yield. The high grain yield observed in the best crosses can be explained by the favorable combination of the alleles in the parents. These finding suggested that it is possible to obtain soybean genotype having good characters required on seed yield and for specific field condition such as drought stress to anticipate the climate change condition.

## 4. Conclusion

There was a reduction on seed weight/plant due to drought stress compared to normal irrigation water condition. In drought condition, line (G/D) -99-32-14 had the highest seed weight/plant, highest drought STI, highest heterosis value, and highest heterobeltiosis value base on seed weight/plant. In drought condition, most of the soybean lines showed positive heterosis values on 100 seed weight, suggested that these lines had 100 seed weight higher than the midparent (Dering 1 and Grobogan). In contrary, all of soybean lines showed negative heterobeltiosis values, suggested that there was no soybean line had 100 seed weight higher than the better parent (Grobogan). The cross of Dering 1 and Grobogan varieties produced one soybean line (G/D) -99-32-14 which was more tolerant to drought and has seed size (100 seed weight) higher than Dering 1 (the current drought tolerance variety). These findings suggested that it is possible to obtain soybean genotype having a higher seed yield than the better parent and tolerant to drought.

## Figures and Tables

**Table 1:** Characteristics of Grobogan variety and Dering 1 variety as crossing parents.

No.	Characteristics		Grobogan variety	Dering 1 variety
1	Year of released	:	2008	2012
2	Origin of population	:	Local Malabar Grobogan	Davros × MLG 2984
3	Days of maturity (days)	:	76	81
4	Plant height (cm)		50–60	57
5	Weight of 100 seeds (g)	:	18.0	10.7
6	Seed yield average (t/ha)	:	2.77	2.00
7	Seed yield potential (t/ha)	:	3.40	2.80
8	Adaptation		Irrigated lowland	Rainfed lowland and upland
9	Drought response in the reproductive phase	:	Susceptible	Tolerant

*Note:* Source: Musadad et al. [9].

**Table 2:** Drought stress tolerant index, heterosis, and heterobeltiosis base on seed weight/plant of soybean genotypes in two irrigation conditions.

No.	Genotypes	Seed weight/plant			Heterosis^a^			Heterobeltiosis^a^		
		Normal (g)	Drought (g)	STI^b^	Normal (%)	Drought (%)	STI (%)	Normal (%)	Drought (%)	STI^b^ (%)
1	(G/D)-75-30-12	14.25	5.13	0.57	62	−25	18	54	−39	−5
2	(G/D)-75-50-30	12.39	7.97	0.77	41	17	60	34	−5	28
3	(G/D)-88-31-13	12.03	7.16	0.67	37	5	40	30	−15	12
4	(G/D)-94-9-4	9.60	7.03	0.52	9	3	9	4	−16	−13
5	(G/D)-97-10-5	13.15	7.53	0.77	50	10	60	42	−10	28
6	(G/D)-99-32-14	11.70	9.48	0.86	33	39	80	26	13	44
7	(G/D)-100-33-15	12.91	7.74	0.78	47	13	62	40	−8	30
8	(G/D)-102-34-16	11.59	7.92	0.71	32	16	49	25	−6	19
9	(G/D)-235-42-23	11.11	8.53	0.74	27	25	54	20	2	23
10	(G/D)-239-43-24	9.88	5.43	0.42	13	−20	−13	7	−35	−30
11	(G/D)-240-44-25	10.83	8.40	0.71	23	23	47	17	0	18
12	(G/D)-241-45-26	10.07	4.87	0.38	15	−29	−21	9	−42	−36
13	(G/D)-242-46-27	11.82	4.78	0.44	35	−30	−8	28	−43	−27
14	(G/D)-245-47-28	10.85	6.90	0.58	24	1	21	17	−18	−3
15	(G/D)-257-48-29	13.00	6.37	0.64	48	−7	34	41	−24	7
16	Dering (D)^c^	9.25	8.40	0.60	—	—	—	—	—	—
17	Grobogan (G)^c^	8.31	5.26	0.34	—	—	—	—	—	—
Mean		11.34	6.99	0.62	33.0	2.7	32.8	26.3	−16.5	6.2

^a^Heterosis (%) and heterobeltiosis (%) base on seed weight/plant.

^b^STI = stress tolerant index.

^c^Parents of 15 soybean lines: Dering 1 variety = tolerant drought but small seed size and Grobogan variety = large seed size but susceptible to drought.

**Table 3:** Drought stress tolerant index, heterosis, and heterobeltiosis base on 100 seeds weight of soybean genotypes in two irrigation conditions.

No.	Genotypes	100 seeds weight			Heterosis^a^			Heterobeltiosis^a^		
		Normal (g)	Drought (g)	STI^b^	Normal (%)	Drought (%)	STI^b^ (%)	Normal (%)	Drought (%)	STI^b^ (%)
1	(D/G)-75-30-12	17.33	13.31	0.94	3	16	11	−21	−8	−27
2	(D/G)-75-50-30	17.06	12.50	0.86	1	9	3	−22	−13	−32
3	(D/G)-88-31-13	15.02	12.78	0.78	−11	11	−7	−32	−11	−39
4	(D/G)-94-9-4	14.97	12.93	0.78	−11	13	−7	−32	−10	−39
5	(D/G)-97-10-5	16.05	13.24	0.86	−5	15	3	−27	−8	−33
6	(D/G)-99-32-14	15.85	13.49	0.87	−6	18	3	−28	−6	−32
7	(D/G)-100-33-15	14.86	13.38	0.81	−12	17	−4	−32	−7	−37
8	(D/G)-102-34-16	15.94	12.77	0.83	−5	11	−2	−27	−11	−36
9	(D/G)-235-42-23	14.08	11.28	0.64	−16	−2	−23	−36	−22	−50
10	(D/G)-239-43-24	15.47	13.66	0.86	−8	19	2	−29	−5	−33
11	(D/G)-240-44-25	14.76	13.58	0.81	−12	18	−3	−33	−6	−37
12	(D/G)-241-45-26	16.70	11.56	0.78	−1	1	−7	−24	−20	−39
13	(D/G)-242-46-27	15.58	11.89	0.75	−8	4	−11	−29	−17	−41
14	(D/G)-245-47-28	15.99	14.53	0.94	−5	27	12	−27	1	−26
15	(D/G)-257-48-29	15.26	13.11	0.81	−9	14	−3	−30	−9	−37
16	Dering (D)^c^	11.77	8.52	0.41	—	—	—	—	—	—
17	Grobogan (G)^c^	21.93	14.41	1.28	—	—	—	—	—	—
Mean		15.71	12.73	0.82	−8.0	12.5	−3.2	−29.1	−10.4	−36.5

^a^Heterosis (%) and heterobeltiosis (%) base on 100 seeds weight.

^b^STI = stress tolerant index.

^c^Parents of 15 soybean lines: Dering 1 variety = tolerant drought but small seed size and Grobogan variety = large seed size but susceptible to drought.

**Table 4:** Stress tolerant index and heterosis of normal pods number/plant of soybean genotypes in two irrigation conditions.

No.	Genotypes	Normal pods number/plant			Heterosis of normal pods number/plant^a^			Empty pods number/plant		
		Normal (g)	Drought (g)	STI^b^	Normal (%)	Drought (%)	STI^b^ (%)	Normal (%)	Drought (%)	STI^b^
1	(D/G)-75-30-12	45.9	28.3	0.75	23	−16	−3	4.1	15.9	4.07
2	(D/G)-75-50-30	43.5	32.6	0.82	17	−3	6	3.0	16.4	3.08
3	(D/G)-88-31-13	45.3	36.2	0.95	22	8	22	3.5	11.3	2.47
4	(D/G)-94-9-4	38.5	34.3	0.77	3	2	−2	4.1	7.4	1.90
5	(D/G)-97-10-5	45.5	39.4	1.04	22	17	33	3.7	7.6	1.76
6	(D/G)-99-32-14	43.6	45.2	1.14	17	34	47	3.7	6.8	1.57
7	(D/G)-100-33-15	44.0	42.0	1.07	18	25	38	3.7	6.2	1.43
8	(D/G)-102-34-16	39.7	41.9	0.97	7	25	24	3.8	8.5	2.02
9	(D/G)-235-42-23	42.9	45.5	1.13	15	35	45	3.9	6.7	1.63
10	(D/G)-239-43-24	37.3	23.3	0.50	0	−31	−35	3.6	18.5	4.16
11	(D/G)-240-44-25	39.7	42.1	0.97	7	25	24	4.7	6.6	1.94
12	(D/G)-241-45-26	38.7	21.7	0.49	4	−36	−37	3.4	20.5	4.36
13	(D/G)-242-46-27	44.3	28.5	0.73	19	−15	−6	2.8	14.1	2.47
14	(D/G)-245-47-28	37.4	31.9	0.69	1	−5	−11	3.3	13.3	2.74
15	(D/G)-257-48-29	45.5	30.9	0.82	22	−8	5	3.9	14.4	3.51
16	Dering (D)^c^	44.2	47.2	1.21	—	—	—	3.2	8.5	1.70
17	Grobogan (G)^c^	30.2	20.1	0.35	—	—	—	9.5	19.5	11.58
Mean		41.5	34.8	0.84	12	3	8	4.0	11.9	2.97

^a^Heterosis (%) base on normal pods number/plant.

^b^STI = stress tolerant index.

^c^Parents of 15 soybean lines: Dering 1 variety = tolerant drought but small seed size and Grobogan variety = large seed size but susceptible to drought.

## Data Availability

The data used to support the findings of this study are available from the corresponding author upon request.
